# Plain Language Summary on Scientific opinion on the tolerable upper intake level for manganese

**DOI:** 10.2903/j.efsa.2023.p211201

**Published:** 2023-12-08

## Abstract

This publication is linked to the following EFSA Journal article: https://efsa.onlinelibrary.wiley.com/doi/10.2903/j.efsa.2023.8413

## Background to the scientific opinion

- Risk managers need advice on the safety of nutrients to establish maximum levels that can be allowed in food supplements and for food fortification purposes, among others.
- For nutrients, no risk of adverse effects is expected unless a threshold intake is exceeded. The identification of this threshold is used as a basis to establish a tolerable upper intake level (UL).
- Excess dietary intake of manganese can lead to adverse health effects, such as neurotoxicity.
- In 2000, the Scientific Committee on Food (SCF) could not set a UL for manganese due to limited available evidence in humans and the lack of a no observed adverse effect level (NOAEL) from animal studies. The SCF however concluded that oral exposure to manganese beyond that normally present in food and beverages could represent a risk of adverse health effects.

## What was EFSA asked to do?

- The European Commission requested a review of the scientific opinion of the SCF based on newly available evidence.
- If there were insufficient data to determine a UL for manganese, EFSA was asked to advise on the "maximum amount that EFSA can confidently conclude poses no risk of adverse effects in the general population," referred to as a 'safe level of intake'.
- Both ULs and safe levels of intake are meant to protect consumers against potential adverse effects related to excess nutrient intakes. The distinction relates to the different way in which these values are established.
- As opposed to ULs, safe levels of intake are not based on an identified threshold of intake above which the risk of adverse intake starts to increase.
    - Intake levels above the safe levels of intake do not necessarily mean that there is a risk of adverse effect.
    - These levels cannot be used to characterise the proportion of the population at risk of adverse effects.

## How did EFSA carry out this work?

- EFSA carried out systematic literature reviews of data on humans and animals to assess evidence regarding excess manganese intake.
- In accordance with the Guidance of the EFSA Panel on Nutrition, Novel Foods and Food Allergens (NDA, 2022), the following questions were addressed:
    - What is the maximum level of total chronic daily intake of manganese (from all sources) which is not expected to pose a risk of adverse health effects to humans?
    - What is the daily intake of manganese from all dietary sources in EU populations?
    - What is the risk of adverse effects related to the intake of manganese in EU populations, including attendant uncertainties?
- Evidence was reviewed to outline:
    - absorption, distribution, metabolism and excretion (ADME) of manganese;
    - biomarkers of exposure to manganese;
    - neurotoxicity of manganese;
    - other adverse health effects.
- Manganese intakes from natural sources were estimated using the EFSA Comprehensive European Food Consumption Database and data on the consumption of manganese from food supplements and fortified foods were collected from national reports on food consumption surveys.

## What were the limitations/uncertainties?

- Neurotoxicity is a well-established adverse effect of excess manganese exposure. However, data to identify critical intakes associated with increased risks of neurotoxicity are lacking both in animals and humans.
- Regarding human studies:

- Most evidence indicated associations between manganese concentrations in drinking water (which typically accounts for a relatively low portion of the total manganese dietary exposure) and neurological adverse effects.
  - Studies investigating the association between total manganese dietary exposure and neurological adverse effects are scarce.
  - Robust conclusions could not be drawn, due to methodological limitations.
- Regarding animal studies:
  - Most studies were not designed to investigate the dose-response relationship between dietary exposure to manganese and neurological effects.
  - As such, the studies were not suitable for use in determining a manganese UL in humans.

## What were the outcomes and their implications?

- Data from available studies are still not adequate to define a reference point, and so no UL for manganese could be established.
- EFSA therefore established a safe level of intake based on the observed intake data of high consumers of manganese in European populations.
- These safe levels of intake are:
  - 8 mg/day for adults (including pregnant and lactating women); and
  - between 2 and 7 mg/day for other population groups.
- These levels refer to the overall manganese intake from all dietary sources, including water, fortified foods and supplements.
- The established safe levels of intake for manganese are based on a conservative approach and are deemed appropriate until adequate data become available to specify the manganese UL.
- For high consumers of manganese from natural sources, the potential risk of adverse effects related to additional consumption of manganese from other sources (e.g. fortified foods and/or food supplements) remains unknown.

## What are the key recommendations?

Recommendation for policy makers and risk managers:

- In place of a UL, safe levels of intake should be used by risk managers to provide guidance on intake levels that can be considered as ‘safe’ for different age groups.

Recommendations for the research community:

- Several data gaps that warrant further investigation and research have been identified that may help lead to the determination of a future UL for manganese:
  - establishing a reliable biomarker of exposure to manganese;
  - investigating the association between overall manganese intake (i.e. from all dietary sources) and adverse effects in human populations;
  - adequate animal toxicity studies which could be used to derive a UL for manganese in humans.

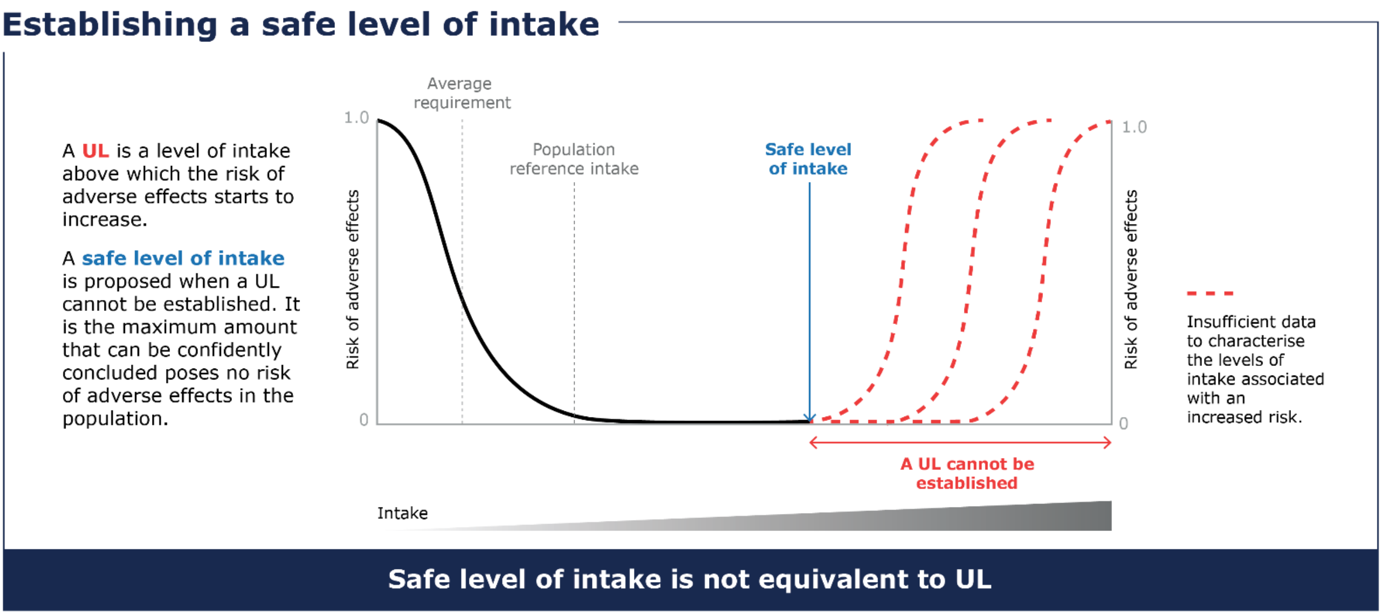

**Figure: Illustration of the difference between the safe level of intake and the UL for a nutrient**

## Glossary

Biomarker of exposure: Chemicals, or chemical metabolites, that can be measured in the body or after excretion from the body and are used to determine an organism's exposure to a substance.

No observed adverse effect level (NOAEL): The greatest concentration or amount of a substance at which no detectable adverse effects occur in an exposed population.

Reference point (also called a point of departure): The point of a dose–response, established from experimental data, used to derive a UL. A reference point can be identified from animal or human data.

Tolerable upper intake level (UL): The maximum daily intake of a nutrient which is not expected to pose a risk of adverse health effects to humans.

**Disclaimer**

- This plain language summary (PLS) is a simplified communication of EFSA's *Scientific opinion on the Tolerable Upper Intake Level for Manganese*. The full EFSA opinion can be found here.
- The purpose of the PLS is to enhance transparency and inform interested parties on EFSA's work on the topic using simplified language to present a summary of the main findings.

EUROPEAN FOOD SAFETY AUTHORITY

The EFSA Journal is a publication of the European Food Safety Authority, an agency of the European Union
